# An unusual site of gallstones five years after laparoscopic cholecystectomy

**DOI:** 10.1016/j.ijscr.2019.02.038

**Published:** 2019-03-05

**Authors:** Sean Heywood, Ben Wagstaff, Noel Tait

**Affiliations:** a Department of General Surgery, Canberra Hospital, Yamba Drive, Garran, ACT, 2615, Australia; b Department of General Surgery, Moruya District Hospital, 2 River Street, Moruya, NSW, 2537, Australia

**Keywords:** Gallstones, Spillage, Complication, Laparoscopic cholecystectomy

## Abstract

**Introduction:**

Gallstone spillage during laparoscopic cholecystectomy is a common occurrence. Complications due to spilled gallstones occur in up to 5% of laparoscopic cholecystectomy cases, with complications having been reported up to 20 years after laparoscopic cholecystectomy.

**Case Report:**

We report the case of a 70 year old male who presented for elective right inguinal hernia repair. At the time of repair he was found to have multiple foreign bodies embedded within an indirect hernia sac. Subsequent pathology confirmed these to be gallstones, having been spilled during emergency laparoscopic cholecystectomy 5 years prior.

**Discussion:**

Gallbladder perforation and gallstone spillage during laparoscopic cholecystectomy occurs frequently, complications due to spilled gallstones are estimated to occur in less than 5% of cases. The most common complications is abscess formation and infection. Though spilled gallstones have been implicated in the formation of colocutaneous, colovesical, and biliocutaneous fistulae. Following gallbladder perforation during cholecystectomy, closure of the hole should be attempted with laparoscopic graspers, surgical clips, or a laparoscopic ligature. Meticulous collection of all visible spilled gallstones should then take place, followed by intraperitoneal lavage ensuring care is taken not to disperse gallstones throughout the peritoneal cavity.

**Conclusion:**

Gallbladder perforation during in laparoscopic cholecystectomy is common. Prevention of gallstone spillage, and retrieval of spilled gallstones is essential in minimising the risk of complications due to spillage.

## Highlights

• Gallbladder perforation in gallstone spillage during laparoscopic cholecystectomy is common.
• Complications due to spilled gallstones occur in up to 5% of cases.
• Complications can be delayed for several years.
• Spilled gallstones should retrieved with all effort to avoid future morbidity.

## 1 Introduction

Laparoscopic cholecystectomy is one of the most commonly performed operations by general surgeons, with approximately 50,000 taking place in Australia annually [1]. Iatrogenic gallbladder perforation and spillage of gallstones during laparoscopic cholecystectomy is a frequent occurrence with rates reported between 1.4 and 40% [2]. There are many reported risk factors for iatrogenic gallbladder perforation during laparoscopic cholecystectomy including male sex, acute cholecystitis, chronic cholecystitis with thickened gallbladder wall >7 mm, and previous laparotomy [3,4]. Iatrogenic gallbladder perforation, with or without gallstone spillage, has been shown to be independently associated with higher rates of surgical site infections and longer hospital stays [5].

This case report has been reported in line with the SCARE criteria [6].

## 2 Case

We present the case of a 70 year old male who had an elective right inguinal hernia repair. He reported a longstanding history of a right inguinal lump which had been causing increasing discomfort over the previous 12 months. His past medical history included an emergency laparoscopic cholecystectomy 5 years prior, as well as atrial fibrillation. On examination, the patient had a mildly tender right inguinoscrotal hernia. Despite being tender, the hernia was reducible and there were no overlying skin changes. Abdominal and testicular examinations were otherwise unremarkable.

**Figs. 1 and 2:**
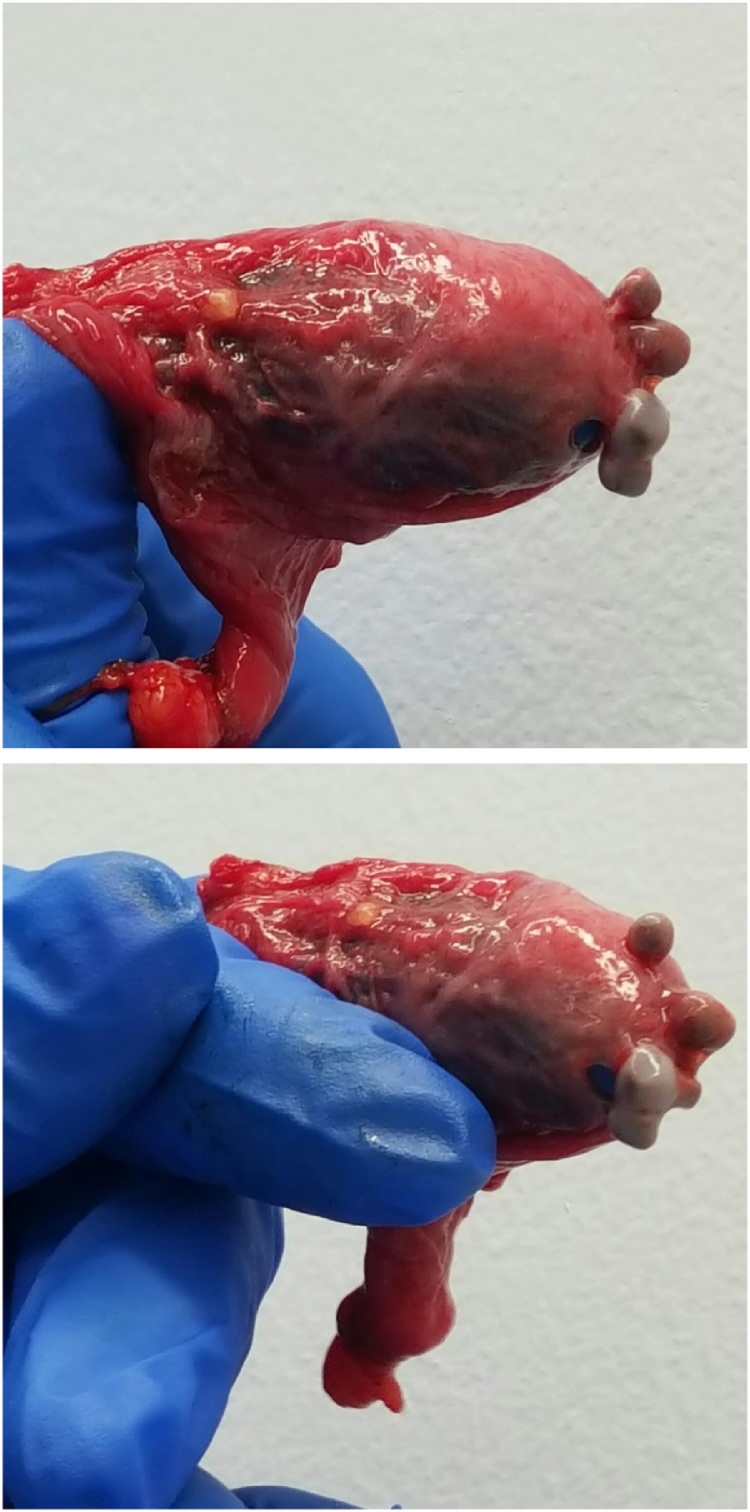
Photo of excised right inguinal sac with four 5 mm gallstones within the hernia sac wall.

Open right inguinal hernia repair was performed using a modified Kugel technique. Intraoperative findings validated clinical examination and a large indirect hernia was reduced. Upon reduction, the hernia sac was found to have multiple 5 mm foreign bodies embedded into the wall. On closer inspection these foreign bodies were macroscopically consistent with gallstones (see Figs. 1 and 2, Figs. 1 and 2). The hernia sac and foreign bodies were sent to the pathologist who confirmed the foreign bodies to be cholesterol gallstones.

The immediate post-operative recovery was uneventful and the patient was discharged home the following day. He was reviewed four weeks later in the outpatient surgical clinic where he reported a good recovery.

## 3 Discussion

Despite the frequency of gallstone spillage, complications secondary to spilled gallstones are rare. In 2015, Demirbas and colleagues reviewed nine studies of 500 or more laparoscopic cholecystectomy cases that reported on gallbladder perforation and gallstone spillage. They observed that the rate of complications due to spilled gallstones ranged between 0–4.7%. Given spilled gallstones can cause complications up to 20 years after laparoscopic cholecystectomy it is possible that these studies underestimate the rate of complications from gallstone spillage due the limited follow up period in some of the studies (mean range 22–121 months) [2,7]. Spilled gallstones have been reported to be associated with abscesses. These are most commonly intraperitoneal, at port sites, or within the thorax. It has been commonly reported to be associated with fistulae, including colocutaneous, colovesical, and biliocutaneous fistulae. More unusual reported complications include infertility, middle colic vessels thrombosis, and acute appendicitis [2]. Similar to our case, gallstones have been reported twice previously within inguinal hernia sacs several years after laparoscopic cholecystectomy [8,9]. In addition to causing complications, spilled gallstones can also mimick peritoneal implants and nodules. This can lead to unnecessary invasive investigation, as well as undue stress and anxiety to patients and their families [9].

Given that the rate of complication due to spilled gallstones during in laparoscopic cholecystectomy can be as high as 5%, [7] there has been much discussion about the appropriate techniques to prevent gallbladder perforation and gallstone retrieval in the event of spillage.^2,11^ Conversion to open cholecystectomy for management of gallstone spillage has been to some extent controversially advocated for, but most recommendations now are to complete the operation laparoscopically. In the event of a gallbladder perforation, closure of the hole should be attempted with laparoscopic graspers, surgical clips, or a laparoscopic ligature. This is to assist in completion of the gallbladder dissection without further spillage. Once a spillage has occurred, meticulous collection of all visible gallstones should take place, and washout and suction should be performed in order to retrieve further stones. Special care should be taken not to disperse stones through the peritoneal cavity when using irrigation.^11^

## 4 Conclusion

This case demonstrates the frequency in which gallstones are spilled during laparoscopic cholecystectomy and that gallstones can remain unabsorbed for long periods of time in ectopic locations. This highlights the importance of meticulous retrieval of spilled gallstones to minimise the risk of associated morbidity.

## Conflict of interest statement

None declared

## Funding

This research did not receive any specific grant from funding agencies in the public, commercial, or not-for-profit sectors.

## Ethical approval

Not applicable. The study is exempt from ethical approval in our institution.

## Consent

Written informed consent was obtained from the patient for publication of this case report and accompanying figures.

## Author contribution

Sean Heywood – Writing original draft, review and editing

Benjamin Wagstaff – Writing original draft, review and editing

Noel Tait – conceptualisation, supervision

## Registration of research studies

Not applicable

## Guarantor

Sean Heywood and Benjamin Wagstaff
